# Risk of Environmental Exposure to H7N9 Influenza Virus *via* Airborne and Surface Routes in a Live Poultry Market in Hebei, China

**DOI:** 10.3389/fcimb.2021.688007

**Published:** 2021-06-07

**Authors:** Cheng Zhang, Kangkang Guo, Huan Cui, Ligong Chen, Chunmao Zhang, Xuejing Wang, Jiaming Li, Yingying Fu, Zhongyi Wang, Zhendong Guo, Juxiang Liu, Shishan Dong

**Affiliations:** ^1^ College of Veterinary Medicine, Hebei Agricultural University, Baoding, China; ^2^ Changchun Veterinary Research Institute, Chinese Academy of Agriculture Sciences, Changchun, China; ^3^ College of Animal Medicine, Jilin University, Changcchun, China; ^4^ Beijing Institute of Biotechnology, Beijing, China

**Keywords:** H7N9 virus, pathogenicity, environmental and airborne transmissibility, mammalian adaption, human exposure risk

## Abstract

Environmental transmission of viruses to humans has become an early warning for potential epidemic outbreaks, such as SARS-CoV-2 and influenza virus outbreaks. Recently, an H7N9 virus, A/environment/Hebei/621/2019 (H7N9), was isolated by environmental swabs from a live poultry market in Hebei, China. We found that this isolate could be transmitted by direct contact and aerosol in mammals. More importantly, after 5 passages in mice, the virus acquired two adaptive mutations, PB1-H115Q and B2-E627K, exhibiting increased virulence and aerosol transmissibility. These results suggest that this H7N9 virus might potentially be transmitted between humans through environmental or airborne routes.

## Introduction

The emergence of H7N9 avian influenza virus (AIV) has drawn the attention of the public due to its high mortality (Tanner et al., 2015). Since its emergence, AIV has spread worldwide (Zhu et al., 2016) and not only causes considerable economic losses but also threatens public health. H7N9 was first detected in 2013 in sick people in Shanghai and Anhui, China (Vidana et al., 2018). Subsequently, some human cases of H7N9 were reported in other cities of China. From 2013 to 2016, the H7N9 AIVs isolated from patients in China had low pathogenicity, but in 2017, highly pathogenic H7N9 AIVs were isolated from patients (Zhang et al., 2013; Shi et al., 2018). The fifth epidemic wave became particularly serious in fall 2016. From October 2016 to June 2017, 750 human cases of H7N9 AIV were reported nationwide, with 282 deaths (Ma et al., 2020).

According to previous studies, the internal genes of H7N9 AIV are derived from genetic recombination of viral subtypes in wild birds in northeast Asia and chickens in Shanghai, Zhejiang and Jiangsu Provinces of China, indicating that H7N9 is a new multicomponent recombinant virus (Su et al., 2015; Tang and Wang, 2017; Yang et al., 2017). The HA gene of H7N9 shares high homology with that of H7N3, while the NA gene shares high homology with the NA genes of H4N9 and H11N9 (Gao et al., 2013). The traits limiting AIV transmission in mammals are polygenic, and overcoming these limitations requires the adaptation of HA, NA, and the RNA polymerase subunit PB2 (Zhang et al., 2017a). Recent studies found that the H7N9 AIV has adapted to mammals through many mutations. Sha et al. reported that mutations in PB2 (E627K), NA (R294K) and PA (V100A) were significantly correlated with increased mortality, while other mutations in HA (N276D) and PB2 (N559T) were distinctly correlated with mild cases (Gao et al., 2009; Wu et al., 2013; Li et al., 2014; Hu et al., 2016; Sha et al., 2016). Overall, the transmissibility of H7N9 to mammals and the lack of pre-existing immunity to H7N9 in humans suggested that H7N9 viruses might pose a potential pandemic threat to humans through further mammalian adaptation.

Hence, the molecular features involved in the adaptation of H7N9 viruses to mammals should be further elucidated. Recently, we isolated an H7N9 AIV strain by environmental swabs from a live poultry market in Hebei, China. The isolate was experimentally evaluated to determine whether it could potentially transmit between humans through environmental or airborne routes. We found that this isolate could be transmitted by direct contact and aerosol in mammals. More importantly, after 5 passages in mice, the virus acquired two adaptive mutations, PB1-H115Q and B2-E627K, exhibiting increased virulence and aerosol transmissibility. These results suggest that this H7N9 virus might potentially be transmitted between humans through environmental or airborne routes.

## Materials And Methods

### Ethics Statement

All animals were adequately cared for, and the animal studies were conducted in strict accordance with the guidelines of animal welfare of the World Organization for Animal Health. Experimental protocols involving animals were approved by the Animal Care and Use Committee of Military Veterinary Institute. All experiments with the influenza A (H7N9) viruses were performed in biosafety level 3 (BSL-3) laboratories approved by the Academy of Military Medical Sciences.

### Virus Isolation

The H7N9 AIV A/environment/Hebei/621/2019 (H7N9) (abbreviated WT) (GenBank: MW433850 and MW433851) was isolated in January 2019 from environmental swabs at a live poultry market in Hebei, China. The virus stocks were grown in specific-pathogen-free (SPF) chicken eggs and maintained at −80°C. The WT strain was isolated from cotton swabs of surfaces at a live poultry market. After sample collection, the swabs were stored in 1 mL Dulbecco’s modified Eagle’s medium (DMEM) containing 2% penicillin-streptomycin antibiotic solution. The tubes containing cotton swabs were swirled by a vortex oscillator for 30 s and then centrifuged at 8000 ×g for 5 min. After centrifugation, 200 μL of the supernatant was incubated in 9-day-old chicken embryos at 37°C. With the same incubation method, the allantoic fluid was passed for five generations, and the allantoic fluid of the fifth generation was harvested. Quantitative RT-PCR was performed by using specific primers from a previous study to determine whether the virus was isolated (Hoffmann et al., 2001). The MA-P5 virus is a mouse-adapted strain generated in this study. The mouse-adapted MA virus was isolated from a series of continuous H7N9 AIV lung-to-lung passages in mice. Briefly, three 6-week-old female BALB/c mice were inoculated intranasally with 50 μL of 10^6^ 50% egg infectious dose (EID_50_) H7N9 subtype AIV after ether anesthesia. Then, 72 h post infection (hpi), the lung tissues of the infected mice were collected and homogenized, and the lung tissue supernatant was collected by centrifugation. Three 6-week-old female BALB/c mice were again inoculated with 50 μL of the supernatant, and the mouse-adapted H7N9 influenza virus was obtained after 5 passages in the mice. Full-length sequencing analysis was performed for both the isolated virus (WT) and the mouse-adapted strain (MA-P5).

### Animals

Hartley strain female guinea pigs weighing 300 to 350 g and six-week-old female BALB/c mice used in this study were purchased from Merial Vital Laboratory Animal Technology Company. The animals in this experiment were not infected with influenza virus, the influenza serum test was negative, and they were all housed at the BSL-3 facility.

### Sequence Analysis

Viral RNA (vRNA) was extracted from virus-infected allantoic fluid, and cDNA was synthesized from vRNAs by reverse transcription with Uni12 primers and PCR amplified with gene-specific primers. Viral gene segments were sequenced by the Comate Bioscience Company (Changchun, China) (Hoffmann et al., 2001). DNA sequences were analyzed by using the Lasergene sequence analysis software package (DNASTAR, Madison, WI).

### Receptor-Binding Specificity Assay

**Figure 1:**
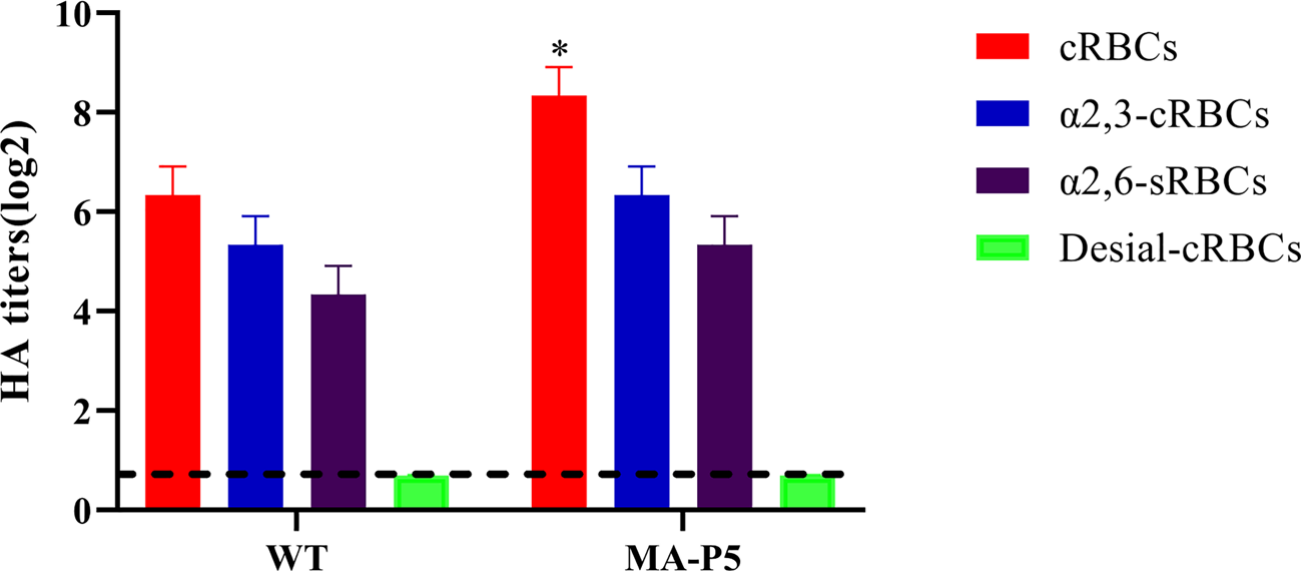
The agglutination activities of the WT and MA-P5 viruses in three types of chicken red blood cells (cRBCs) and sheep red blood cells (sRBCs) were determined by HA titer. cRBCs, cRBCs with α-2,3-linked sialic acid receptors and α-2,6-linked sialic acid receptors; α-2,3 cRBCs, cRBCs treated with α-2,3-sialidase (with only α-2,6-linked sialic acid receptors); α-2,6 sRBCs, sRBCs (with only α-2,3-linked sialic acid receptors); and desialylated (desial) cRBCs, cRBCs treated with VCNA (no receptors). The values are shown as the means and standard deviations of three independent experiments. *P < 0.05 (MA-P5 vs WT).

The receptor-binding specificities of human influenza viruses were determined by HA assays with 1% chicken red blood cell (cRBC) and sheep RBC (sRBC) suspensions. A similar approach was used in our previous study (Zhao et al., 2019). Four types of RBCs were used: cRBCs, cRBCs with both α-2,3-linked sialic acid receptors and α-2,6-linked sialic acid receptors; α-2,3 cRBCs, cRBCs treated with α-2,3-sialidase (with only α-2,6-linked sialic acid receptors); α-2,6 sRBCs, sRBCs with only α-2,3-linked sialic acid receptors; and desialylated (desial) cRBCs, cRBCs treated with *Vibrio cholera* neuraminidase (VCNA) (no receptors). For sialidase treatment, 90 μL of a 10% cRBC suspension was treated with 10 μL of α-2,3-sialidase (50 mU/μL) (TaKaRa, Dalian, China) for 10 min at 37°C. The sample was then washed two times with phosphate-buffered saline (PBS), centrifuged at 1500 rpm for 5 min each time, adjusted to a final working concentration (1%) with PBS, and stored at 4°C. For VCNA (Roche, San Francisco, CA, USA) treatment, 90 μL of a 10% cRBC suspension was treated with 10 μL of VCNA (50 mU/μL) for 1 h at 37°C, washed two times with PBS, centrifuged at 1500 rpm for 5 min each time, adjusted to a final working concentration (1%) with PBS, and stored at 4°C. For the HA assay, viruses were serially diluted 2-fold with 50 μL of PBS and mixed with 50 μL of a 1% RBC suspension in a 96-well plate. HA titers were read after 20 min of the reaction at room temperature. We conducted statistics and analysis on the data in **Figure 1** by using the unpaired t-test.

### Cell Culture and Growth Curves

Madin-Darby canine kidney (MDCK) cells were grown in DMEM supplemented with 10% fetal bovine serum plus antibiotics. MDCK cells were seeded on 12-well plates and inoculated at a multiplicity of infection (MOI) of 0.01 A 50% tissue culture infectious dose (TCID_50_) per cell was used to determine the growth kinetics of WT and MA-P5 H7N9 viruses. The inoculum was removed one hour after incubation, and the cells were washed three times with PBS. Then, fresh medium supplemented with 1 mg/mL TPCK-treated trypsin was added. The supernatants containing the virus were collected at 12, 24, 36, 48 and 60 hpi. The viral titer was determined in 9-day-old SPF embryonated chicken eggs, and the EID_50_ values were calculated by the method of Reed–Muench. The growth kinetics data shown were obtained from three independent tests.

### Adaptation of Isolated H7N9 AIV in Mice

The mouse-adapted virus MA was isolated from a series of continuous H7N9 AIV lung-to-lung passages in mice. Briefly, three 6-week-old female BALB/c mice were inoculated intranasally with 50 μL solution containing 10^6^ EID_50_ H7N9 subtype AIV after ether anesthesia. Then, 72 hpi, the lung tissues of the infected mice were collected and homogenized, and the lung tissue supernatant was collected by centrifugation. Three 6-week-old female BALB/c mice were again inoculated with 50 μL of the supernatant, and the mouse-adapted H7N9 influenza virus was obtained after 5 passages in the mice. Previous studies have shown that during the process of mouse adaptation, it is only necessary to ensure that live virus is contained at each passage, and it is not necessary to measure the EID_50_ of each generation. Therefore, each generation of the mice was inoculated with 50 µL of the supernatant of the mouse lung tissue homogenate. Before each passage in mice, chicken embryos were inoculated with the supernatant to verify that the supernatant of the mouse lung tissue homogenate contained live virus (Zhang et al., 2017a).

### Mouse Study

To determine the pathogenicity of the isolated virus (WT) and the mouse-adapted MA-P5 virus in mice, a group of five 6-week-old female BALB/c mice were inoculated intranasally under ether anesthesia with 50 μL of 10^6^ EID_50_ or PBS. The mice were monitored for weight loss and mortality for 14 days. To study the replication of the virus in mice, a group of 20 6-week-old female BALB/c mice were inoculated intranasally under ether anesthesia with 50 μL of 10^6^ EID_50_ of MA-P5 or WT H7N9 virus or PBS. Three animals were randomly selected from each group at 3 and 5 days post infection (dpi) and euthanized to collect the heart, liver, spleen, lung, kidney and brain. After homogenization of the above tissues, the supernatant was inoculated into 9-day SPF chicken embryos to test the viral titer by the Reed–Muench method. To explore the pathological changes in lung tissues of mice infected with WT and MA-P5 viruses, lung tissues were collected at 5 dpi, fixed in 4% formalin solution, cut into pathological tissue sections, and observed under an optical microscope.

### Guinea Pig Study

To study the transmission ability of the WT virus and mouse-adapted MA-P5 virus, first, a group of 3 guinea pigs were inoculated intranasally under ether anesthesia with 200 μL of 10^6^ EID_50_/mL of the WT and MA-P5 viruses. After 24 h, three naive guinea pigs were placed in the same cages. Nasal washes were collected from each guinea pig at 2, 4, 6, and 8 dpi. Viral titers were determined by titration in eggs as described previously. To explore the aerosol transmission ability of the WT virus and mouse-adapted MA-P5 virus, we selected three healthy animals that were inoculated intranasally with 200 μL of 10^6^ EID_50_/mL of the WT and MA-P5 viruses. Each inoculated animal was separately kept in a cage with a partition. Then, after 24 h, three naive animals were matched with the previously inoculated guinea pigs, and each naive guinea pig was placed in a clean, uncontaminated cage adjacent to an infected partner. Nasal washes were collected from each guinea pig at 2, 4, 6, and 8 dpi. Viral titers were determined by titration in eggs as described previously.

### Statistical Analysis

The statistical significance of differences between experimental groups was determined using the Holm-Sidak method with multiple t-tests (one per row). Differences with P values less than 0.05 were considered significant. The error bars indicate the standard deviation.

## Results

### The WT and MA-P5 Viruses Can Bind to Both Avian and Human Receptors

The ability to bind to human receptors has been identified as a major factor in the cross-species transmission of AIV. We evaluated the receptor-binding specificities of the two H7N9 viruses (WT and MA-P5) using an HA assay. The surface of cRBCs contains α-2,3-linked and α-2,6-linked sialic acid receptors. The surface of cRBCs treated with α-2,3-sialidase contained only α-2,6-linked sialic acid receptors. The surface of cRBCs treated with VCNA contained no receptors, while the surface of sRBCs contained only α-2,3-linked sialic acid receptors. As shown in **Figure 1**, all the H7N9 viruses could agglutinate untreated cRBCs and sRBCs but not VCNA-treated cRBCs. As expected, the MA-P5 and WT viruses could bind to both α2,3-cRBCs and α2,6-cRBC receptors, indicating that the WT and MA-P5 viruses could bind to both avian and human receptors. The RBC binding ability of the MA-P5 virus was enhanced compared to that of the WT virus, and the result was statistically significant.

### The Replication Ability of MA-P5 Was Better Than That of WT in MDCK Cells

**Figure 2:**
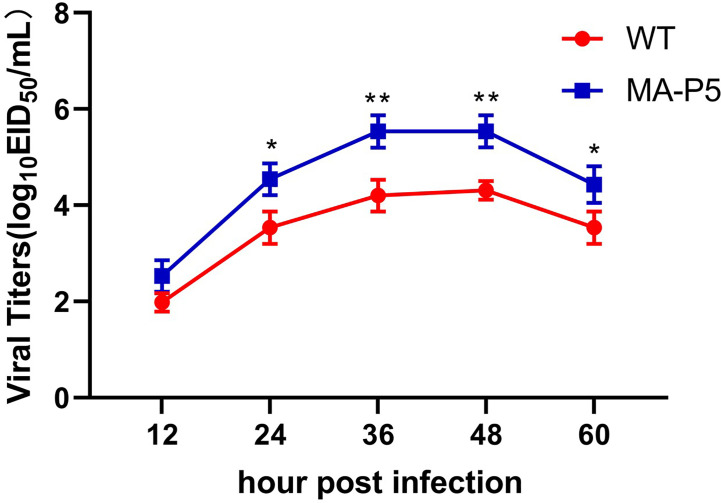
To characterize the growth kinetics, MDCK cells were infected with WT or MA-P5 at an MOI of 0.01 TCID_50_ per cell and treated with 1 µg/mL TPCK. The MDCK cell culture supernatants were harvested at 12, 24, 36, 48 and 60 dpi and stored in a -80°C freezer. The titers were calculated to determine the TCID_50_ at every time point by the Reed–Muench method. *P < 0.05 (MA-P5 vs WT), **P < 0.01 (MA-P5 vs WT). To determine the virulence of WT and MA-P5, mice (n = 5) were intranasally inoculated with 10^6^ EID_50_ of WT and MA-P5. An equal volume of PBS was used as a negative control.

To explore the replication ability of the WT and MA-P5 H7N9 viruses, we also tested the growth curves of the viruses in MDCK cells. The viral titers of WT and MA-P5 similarly reached a peak at 48 hpi, and the titers were 10^4.31^ EID_50_/mL and 10^5.54^ EID_50_/mL, respectively (**Figure 2**). Then, the titers of the WT and MA-P5 viruses in MDCK cells began to decline at 60 hpi. The replication ability of the MA-P5 virus was significantly higher than that of the WT virus, and the titer of the MA-P5 virus was approximately 10-fold higher than that of the WT virus (P < 0.01, n = 3).

### MA-P5 Showed Increased Pathogenicity

**Figure 3:**
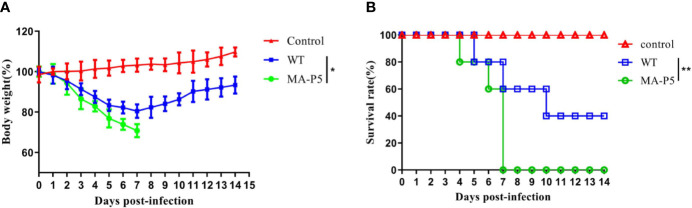
**(A)** The mice in each group were monitored for body weights daily for 14 days. Data shown are the means and standard deviations (SD) for each group. **(B)** The infected mice were observed for 14 consecutive days, and the mortality rate was recorded. *P < 0.05 (MA-P5 vs WT), **P < 0.01 (MA-P5 vs WT).

In the pathogenicity analysis, the MA-P5-inoculated group lost weight rapidly and was down to approximately 75% of the initial body weight at 7 dpi, while the WT-inoculated group lost weight slowly until the weight began to rise at 7 dpi (**Figure 3A**). The median lethal doses (MLD_50_s) of the MA-P5 and WT viruses were 10^4.3^ and 10^5.3^ MLD_50_/mL, respectively. The WT-inoculated mice began to die at 5 dpi, and the survival rate was 40% at 10 dpi, after which no deaths occurred. However, the MA-P5-inoculated mice began to die at 4 dpi, and all died at 7 dpi (**Figure 3B**). Based on the above data, we concluded that MA-P5 was more virulent than the WT virus in mice.

### MA-P5 Showed Greater Replication Ability in Mice

**Figure 4:**
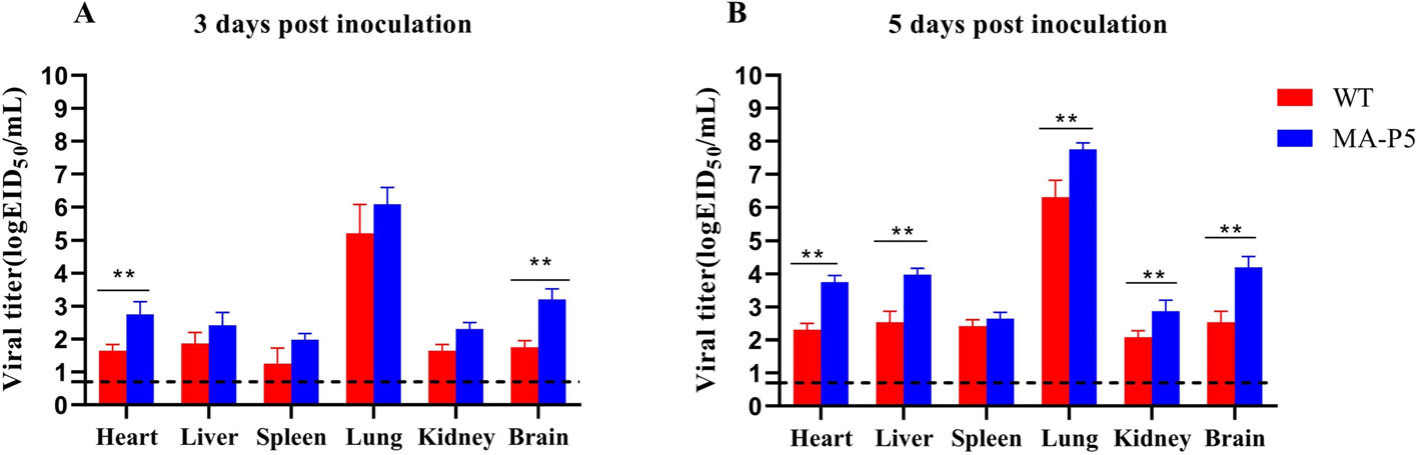
WT and MA-P5 virus replication in the heart, liver, spleen, lung, kidney and brain at 3 dpi **(A)** and 5 dpi **(B)**. The average of each group is shown, and the error bars represent the SD. All data were analyzed by using one-way analysis of variance, **P < 0.01 (MA-P5 vs WT).

**Figure 5:**
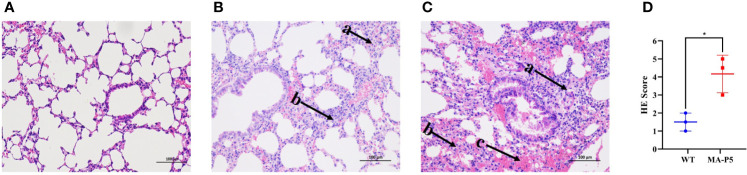
Histopathology of the lungs of mice inoculated with PBS **(A)**, WT **(B)**, or MA-P5 **(C)**. The HE score results are shown in **(D)**. The infected mouse lungs were fixed with formalin, embedded in paraffin and stained with hematoxylin and eosin. Images were obtained at 20× magnification; arrow a, lymphocytes; arrow b, neutrophils; arrow c, bleeding. Pathological severity scores in infected mice, based on the inflammation area percentage for each section of the lungs collected from each animal using the following scoring system: 0, no pathological change; 1, affected area ≤ 10%; 2, affected area < 50% and > 10%; and 3, affected area ≥ 50%; an additional point was added to the score when pulmonary edema and/or alveolar hemorrhage was observed. *P < 0.05 (MA-P5 vs WT).

To study the replication of the WT and MA-P5 viruses in mice, the heart, liver, spleen, lung, kidney and brain were collected at 3 dpi (**Figure 4A**) and **Figure 5** dpi (**Figure 4B**), and we found that both the WT and MA-P5 viruses could be detected in all six tissues at 3 and 5 dpi, with virus content highest in the lungs among all tissues. At 3 dpi, the viral titer was increased only for MA-P5 in the heart and brain. At 5 dpi, although there was no difference in the viral titer in the spleen, the viral titer was increased for MA-P5 in the heart, liver, lung, kidney and brain. The titer of the MA-P5 virus in lung tissue was 10^6.21^ EID_50_/mL at 3 dpi and 10^7.76^ EID_50_/mL at 5 dpi. The titer of the WT virus in lung tissue was 10^5.20^ EID_50_/mL at 3 dpi and 10^6.09^ EID_50_/mL at 5 dpi, which was approximately 10-fold lower than that of the MA-P5 virus. Thus, the MA-P5 virus showed higher replication abilities than the WT virus in mice.

### Pathological Analysis of Lung Tissues in the Infected Mice

The WT- and MA-P5-infected mice showed obvious pathological changes in the histopathological analysis (**Figure 5**). In **Figure 5**, the pathological results were statistically analyzed by using the following standard. Pathological severity scores in infected mice were based on the percentage of inflammatory area for each section of the lungs collected from each animal using the following scoring system: 0, no pathological change; 1, affected area ≤10%; 2, affected area <50% and >10%; and 3, affected area ≥50%; an additional point was added to the score when pulmonary edema and/or alveolar hemorrhage was observed. Based on the above analysis, the pathogenicity of the MA-P5 virus in mice was significantly higher than that of the WT virus. In short, the MA-P5 virus showed enhanced pathogenicity in mice, revealing an adaptation from avian species to mammals.

### Study on the Transmission Ability of the WT and MA-P5 Viruses

**Figure 6:**
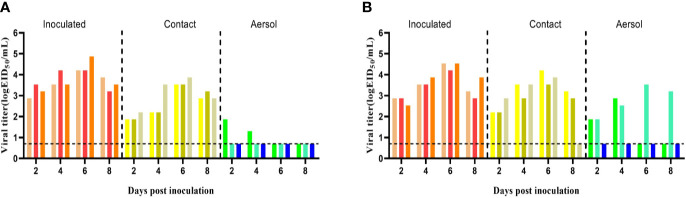
Groups of three guinea pigs seronegative for influenza viruses were inoculated with 10^6^ EID_50_/mL of the WT **(A)** or MA-P5 **(B)** viruses. After 24 h, the three inoculated guinea pigs were individually paired by cohousing with a direct-contact guinea pig; furthermore, an aerosol-contact guinea pig was housed in a wire frame cage adjacent to that of the infected guinea pig. Nasal washes were collected every 2 days continuously for 2, 4, 6, and 8 days. Each color bar represents the viral titer in a single animal. The dashed lines indicate the lower limit of virus detection.

As shown in **Figures 6A, B**, both the WT and MA-P5 viruses could transmit through direct contact and aerosol routes. In the WT aerosol transmission group, viruses could be detected in one guinea pig at 2 and 4 dpi (**Figure 6A**). In the MA-P5 group, viruses could be detected in two guinea pigs at 2 and 4 dpi (**Figure 6B**). In previous studies, transmission analyses were mainly based on the efficiency of different transmission modes (Zhao et al., 2017; Sun et al., 2020). In this study, both the WT and MA-P5 strains had 100% direct contact transmission efficiency (all three guinea pigs in the transmission group were positive). However, the airborne transmission efficiency of WT was 33.3% (one of the three guinea pigs in the transmission group was positive), and the aerosol transmission efficiency of MA-P5 was 66.7% (two of three guinea pigs in the transmission group was positive). Therefore, we concluded that the transmission ability of the MA-P5 strain was enhanced compared to that of the WT strain. These results demonstrated that both WT and MA-P5 could be transmitted by direct contact and aerosols, but the transmission ability of MA-P5 was enhanced.

### Sequence Analysis

The sequence analysis revealed that two amino acid substitutions in the MA-P5 virus, one in the PB1 subunit (H115Q) and another in the PB2 subunit (E627K) (**Table 1**). These two amino acid changes first appeared in the P1 virus and then were stably inherited in the P2, P3, P4, and P5 generations, suggesting that the WT virus quickly adapted to mammals after obtaining the two mutations (**Supplement Table 1**).

**Table 1:** Amino acid substitutions in the mouse-adapted H7N9 influenza virus.

Segment	Position	WT	P1	P2	P3	P4	P5
PB1	115	H	Q	Q	Q	Q	Q
PB2	627	E	K	K	K	K	K

*The WT virus and all passaged H7N9 viruses were sequenced. The nucleotide sequence was translated into protein and used to align the sequence to identify amino acid substitutions. All identified amino acids are listed in the table.

## Discussion

In recent years, a large number of new outbreaks of infectious diseases have originated from cross-species transmission (Zhang et al., 2017b; Guo et al., 2020). Quick adaptions from animals to humans and multiple transmission routes have made it difficult to formulate effective prevention and control measures. In addition to individual transmission, environmental transmission has become a new topic. Since the first case of human infection with H7N9 AIV in China in 2013, H7N9 AIV has also been isolated from poultry, but the virus showed low pathogenicity in poultry. In 2017, the H7N9 AIV acquired a high pathogenicity mutation, and this H7N9 subtype avian influenza, which is highly pathogenic in poultry, is prone to adaptive mutations in mammals. Therefore, the molecular characteristics of H7N9 AIV adapted to mammals must be further studied, and continuous passage in mice has become a common method to determine amino acid changes during the adaptation of influenza viruses to mammalian hosts (Zhao et al., 2017).

The RNA-dependent RNA polymerase (RNP) complex of influenza A virus mediates the transcription and replication of the viral genome. Due to the lack of proofreading ability of this enzyme, the probability of the emergence of mutations during virus replication is high (Feng et al., 2016). The K480R mutation of the PB1 protein may originate from swine flu, and it is found at a higher frequency in swine flu viruses than in human and avian viruses. Studies have found that the K480R mutation of the PB1 protein can cause H5N1 and H1N1 influenza viruses to polymerize in mice, and the increase in enzyme activity is believed to be due to changes in conserved amino acids (Leymarie et al., 2014). The G622D mutation of the PB1 protein of H5N1 AIV weakened the binding of PB1 to vRNA, which significantly reduced the polymerase activity in mice, thereby weakening the virulence of the H5N1 virus. In addition, the N66S mutation of the PB1-F2 protein of influenza A virus increased the virulence of the virus by inhibiting the interferon response in the host at an early stage (Alymova et al., 2011; Chu et al., 2012). This phenomenon specifically manifests as the infiltration of monocytes and neutrophils, which leads to an increase in lung cell numbers and an upregulation of the expression of various cytokines and chemokines related to monocytes and neutrophils, such as MIP-1β, RANTES, IFN-β and IFN-γ (Conenello et al., 2011). Therefore, the amino acid changes in the RNP complex should be given more attention and studied in depth.

In this study, the H7N9 virus was isolated by environmental swabs from a live poultry market in Hebei, China. The ability to bind to human receptors has been identified as a major factor in the cross-species transmission of AIV. As shown in **Figure 1**, MA-P5 and WT can bind to both α2,3-cRBC and α2,6-cRBC receptors, indicating that WT and MA-P5 can bind to both avian and human receptors. These results are indicative of the importance of reinforcing our active surveillance and preparedness to prevent outbreaks of AIVs. In addition, we found that MA-P5 harbored two adaptive mutations and showed increased virulence and enhanced aerosol transmissibility after 5 passages in mice. We identified the E627K mutation in the PB2 gene and the H115Q mutation in the PB1 gene. Compared with control mice, mice infected with MA-P5 lost significantly more weight and had a higher mortality rate. Some studies have shown that an H9N2 strain with the E627K and Q591K mutations in PB2 was more pathogenic in mice than the unmutated H9N2 strain. The mutated strain also increased the number of neutrophils, and an excessive number of neutrophils infiltrated the lungs compared with the unmutated H9N2 strain, which may cause inflammation of the lungs (Kamal et al., 2017). Based on our data, we found that H115Q in PB1 and E627K in PB2 might play an important role in the pathogenesis of the MA-P5 virus in mice. The replication ability of MA-P5 was significantly enhanced *in vitro* and *in vivo* compared to that of WT. In mouse-adapted AIVs, there are many amino acid substitutions, resulting in increased virulence and enhanced replication dynamics *in vitro* and *in vivo*. Studies have confirmed that the enhancement of the polymerase activity of the E627K mutation in the H7N9 PB2 protein was mainly because the PB2 627K residue forms a continuous fundamental plane in the PB2 627K domain, which is very important for RNA binding and polymerase function in mammalian cells. In addition, the transcriptional activity of the recombinant RNP complex containing different substituents was improved, which could contribute to the increased replication efficiency in mammalian cells. Overall, PB1 115Q and PB2 627K of the MA-P5 H7N9 virus may cooperate to enhance virus replication *in vivo* and *in vitro*.

The study on the transmission ability of the WT and MA-P5 viruses in guinea pigs found that MA-P5 could be transmitted by direct contact and airborne routes. Previous studies have shown that E627K of the PB2 gene has a limited effect on the transmission of influenza virus. The airborne transmission ability of MA-P5 might be related to the 115 amino acid change in the PB1 gene from H to Q. Some studies demonstrated that HPAI H5N1 viruses could acquire the ability to transmit efficiently *via* aerosols or respiratory droplets in the ferret model with the presence of 115Q in the PB1 sequences. In addition, we compared the PB1 sequence of MA-P5 with that of human influenza virus, a/Anhui/1/2013 (H7N9), a/Wuxi/4/2013 (H7N9), and a/Jiangsu/03/2013 (H7N9), and found that the 115 amino acids of all were Q.

There have been a large number of reports about the transmission mechanism of AIV among mammals, and the transmission ability of avian influenza is the result of multiple genes. Among them, the HA protein plays an important role in the cross-host transmission of influenza virus (Feng et al., 2016). The MA-P5 virus has the ability to bind to the α-2,3 and α-2,6 sialic acid receptors. It is possible that this receptor-binding property might contribute to the spread of the virus in guinea pigs.

The results of this study suggest that this H7N9 virus might potentially be transmitted between humans through environmental or airborne routes. Constant monitoring of the threat from H7N9 viruses is necessary under different environmental conditions; specifically, the amino acid changes within the RNP complex should be monitored as warnings for H7N9 outbreaks.

## Data Availability Statement

The datasets presented in this study can be found in online repositories. The names of the repository/repositories and accession number(s) can be found below: https://www.ncbi.nlm.nih.gov/genbank/, MW433850 https://www.ncbi.nlm.nih.gov/genbank/, MW433851.

## Ethics Statement

The animal study was reviewed and approved by Animal Care and Use Committee of Military Veterinary Institute.

## Author Contributions

ZW, ZG, JXL, and SD conceived and designed the experiments. ZW, CZ, KG, and HC wrote the manuscript. LC, CMZ, XW, JML, and YF performed some of the experiments. All authors contributed to the article and approved the submitted version.

## Funding

This work was funded by the National Natural Science Foundation of China (No. 3210010512), the Hebei Layer/Broiler Industry Technology System (HBCT2018150210) and Key Research and Development Program of Hebei Province (18227517D). The funders had no role in the study design, data collection and analysis, decision to publish, or preparation of the manuscript.

## Conflict of Interest

The authors declare that the research was conducted in the absence of any commercial or financial relationships that could be construed as a potential conflict of interest.

## References

[B1] Alymova I. V. Green A. M. van de Velde N. McAuley J. L. Boyd K. L. Ghoneim H. E.. (2011). Immunopathogenic and Antibacterial Effects of H3N2 Influenza A Virus PB1-F2 Map to Amino Acid Residues 62, 75, 79, and 82. J. Virol. 85 (23), 12324–12333. 10.1128/jvi.05872-11 21937639 PMC3209399

[B2] Chu C. Fan S. Li C. Macken C. Kim J. H. Hatta M.. (2012). Functional Analysis of Conserved Motifs in Influenza Virus PB1 Protein. PLoS One 7 (5), e36113. 10.1371/journal.pone.0036113 22615752 PMC3352917

[B3] Conenello G. M. Tisoncik J. R. Rosenzweig E. Varga Z. T. Palese P. Katze M. G. (2011). A Single N66S Mutation in the PB1-F2 Protein of Influenza A Virus Increases Virulence by Inhibiting the Early Interferon Response In Vivo. J. Virol. 85 (2), 652–662. 10.1128/jvi.01987-10 21084483 PMC3020033

[B4] Feng X. Wang Z. Shi J. Deng G. Kong H. Tao S.. (2016). Glycine at Position 622 in PB1 Contributes to the Virulence of H5N1 Avian Influenza Virus in Mice. J. Virol. 90 (4), 1872–1879. 10.1128/jvi.02387-15 26656683 PMC4733975

[B5] Gao R. B. Cao B. Hu Y. W. Feng Z. J. Wang D. Y. Hu W. F.. (2013). Human Infection With a Novel Avian-Origin Influenza A (H7n9) Virus. New Engl. J. Med. 368 (20), 1888–1897. 10.1056/NEJMoa1304459 23577628

[B6] Gao Y. Zhang Y. Shinya K. Deng G. Jiang Y. Li Z.. (2009). Identification of Amino Acids in HA and PB2 Critical for the Transmission of H5N1 Avian Influenza Viruses in a Mammalian Host. PLoS Pathog. 5 (12), e1000709. 10.1371/journal.ppat.1000709 20041223 PMC2791199

[B7] Guo Z. D. Wang Z. Y. Zhang S. F. Li X. Li L. Li C.. (2020). Aerosol and Surface Distribution of Severe Acute Respiratory Syndrome Coronavirus 2 in Hospital Wards, Wuhan, Chin. Emerg. Infect. Dis. 26 (7), 1583–1591. 10.3201/eid2607.200885 32275497 PMC7323510

[B8] Hoffmann E. Stech J. Guan Y. Webster R. G. Perez D. R. (2001). Universal Primer Set for the Full-Length Amplification of All Influenza A Viruses. Arch. Virol. 146 (12), 2275–2289. 10.1007/s007050170002 11811679

[B9] Hu M. Chu H. Zhang K. Singh K. Li C. Yuan S.. (2016). Amino Acid Substitutions V63I or A37S/I61T/V63I/V100A in the PA N-Terminal Domain Increase the Virulence of H7N7 Influenza A Virus. Sci. Rep. 6, 37800. 10.1038/srep37800 27886255 PMC5122915

[B10] Kamal R. P. Alymova I. V. York I. A. (2017). Evolution and Virulence of Influenza A Virus Protein Pb1-F2. Int. J. Mol. Sci. 19 (1), 96. 10.3390/ijms19010096 PMC5796046 29286299

[B11] Leymarie O. Embury-Hyatt C. Chevalier C. Jouneau L. Moroldo M. Da Costa B.. (2014). Pb1-F2 Attenuates Virulence of Highly Pathogenic Avian H5N1 Influenza Virus in Chickens. PloS One 9 (6), e100679. 10.1371/journal.pone.0100679 24959667 PMC4069075

[B12] Li X. Shi J. Guo J. Deng G. Zhang Q. Wang J.. (2014). Genetics, Receptor Binding Property, and Transmissibility in Mammals of Naturally Isolated H9N2 Avian Influenza Viruses. PLoS Pathog. 10 (11), e1004508. 10.1371/journal.ppat.1004508 25411973 PMC4239090

[B13] Ma S. Zhang B. Shi J. Yin X. Wang G. Cui P.. (2020). Amino Acid Mutations A286V and T437M in the Nucleoprotein Attenuate H7N9 Viruses in Mice. J. Virol. 94 (2), e01530–19. 10.1128/jvi.01530-19 31666373 PMC6955278

[B14] Sha J. Chen X. Ren Y. Chen H. Wu Z. Ying D.. (2016). Differences in the Epidemiology and Virology of Mild, Severe and Fatal Human Infections With Avian Influenza A (H7N9) Virus. Arch. Virol. 161 (5), 1239–1259. 10.1007/s00705-016-2781-3 26887968 PMC7101734

[B15] Shi J. Z. Deng G. H. Ma S. J. Zeng X. Y. Yin X. Li M.. (2018). Rapid Evolution of H7N9 Highly Pathogenic Viruses That Emerged in China in 2017. Cell Host Microbe 24 (4), 558–55+. 10.1016/j.chom.2018.08.006 30269969 PMC6310233

[B16] Su S. Bi Y. H. Wong G. Gray G. C. Gao G. F. Li S. J. (2015). Epidemiology, Evolution, and Recent Outbreaks of Avian Influenza Virus in China. J. Virol. 89 (17), 8671–8676. 10.1128/Jvi.01034-15 26063419 PMC4524075

[B17] Sun H. Xiao Y. Liu J. Wang D. Li F. Wang C.. (2020). Prevalent Eurasian Avian-Like H1N1 Swine Influenza Virus With 2009 Pandemic Viral Genes Facilitating Human Infection. Proc. Natl. Acad. Sci. U. S. A. 117 (29), 17204–17210. 10.1073/pnas.1921186117 32601207 PMC7382246

[B18] Tang J. Wang D. Y. (2017). Research Progress in Human Infection With Avian Influenza H7N9 Virus. Sci. China-Life Sci. 60 (12), 1299–1306. 10.1007/s11427-017-9221-4 29270791

[B19] Tanner W. D. Toth D. J. Gundlapalli A. V. (2015). The Pandemic Potential of Avian Influenza A(H7N9) Virus: A Review. Epidemiol. Infect. 143 (16), 3359–3374. 10.1017/s0950268815001570 26205078 PMC9150948

[B20] Vidana B. Dolz R. Busquets N. Ramis A. Sanchez R. Rivas R.. (2018). Transmission and Immunopathology of the Avian Influenza Virus A/Anhui/1/2013 (H7N9) Human Isolate in Three Commonly Commercialized Avian Species. Zoonoses Public Health 65 (3), 312–321. 10.1111/zph.12393 28905526

[B21] Wu Y. Bi Y. Vavricka C. J. Sun X. Zhang Y. Gao F.. (2013). Characterization of Two Distinct Neuraminidases From Avian-Origin Human-Infecting H7N9 Influenza Viruses. Cell Res. 23 (12), 1347–1355. 10.1038/cr.2013.144 24165891 PMC3847574

[B22] Yang L. Zhu W. Li X. Chen M. Wu J. Yu P.. (2017). Genesis and Spread of Newly Emerged Highly Pathogenic H7N9 Avian Viruses in Mainland China. J. Virol. 91 (23), e01277–17. 10.1128/jvi.01277-17 28956760 PMC5686710

[B23] Zhang Y. Gong Y. Wang C. Liu W. Wang Z. Xia Z.. (2017b). Rapid Deployment of a Mobile Biosafety Level-3 Laboratory in Sierra Leone During the 2014 Ebola Virus Epidemic. PloS Negl. Trop. Dis. 11 (5), e0005622. 10.1371/journal.pntd.0005622 28505171 PMC5444861

[B24] Zhang Q. Shi J. Deng G. Guo J. Zeng X. He X.. (2013). H7n9 Influenza Viruses Are Transmissible in Ferrets by Respiratory Droplet. Science 341 (6144), 410–414. 10.1126/science.1240532 23868922

[B25] Zhang C. Zhao Z. Guo Z. Zhang J. Li J. Yang Y.. (2017a). Amino Acid Substitutions Associated With Avian H5n6 Influenza A Virus Adaptation to Mice. Front. Microbiol. 8, 1763. 10.3389/fmicb.2017.01763 28966609 PMC5605651

[B26] Zhao Z. Guo Z. Zhang C. Liu L. Chen L. Zhang C.. (2017). Avian Influenza H5n6 Viruses Exhibit Differing Pathogenicities and Transmissibilities in Mammals. Sci. Rep. 7 (1), 16280. 10.1038/s41598-017-16139-1 29176564 PMC5701206

[B27] Zhao Z. Liu L. Guo Z. Zhang C. Wang Z. Wen G.. (2019). A Novel Reassortant Avian H7n6 Influenza Virus Is Transmissible in Guinea Pigs Via Respiratory Droplets. Front. Microbiol. 10, 18. 10.3389/fmicb.2019.00018 30723462 PMC6349713

[B28] Zhu H. Lam T. T. Smith D. K. Guan Y. (2016). Emergence and Development of H7N9 Influenza Viruses in China. Curr. Opin. Virol. 16, 106–113. 10.1016/j.coviro.2016.01.020 26922715

