# Supplementary material for: Risk of Environmental Exposure to H7N9 Influenza Virus via Airborne and Surface Routes in a Live Poultry Market in Hebei, China
Source: Front Cell Infect Microbiol. 2021 Jun 7;11:688007. doi: 10.3389/fcimb.2021.688007 (PMC8216215; doi:10.3389/fcimb.2021.688007)
Supplement: Supplementary file 1 [file DataSheet_1.docx]

>P5-PB1

ATGGATGTCAATCCGACTTTACTTTTCAAAGTGCCAGTACAAAATGCTATAAGTACCACTTTCCCTTATACTGGAGACCCTCCATACAGCCATGGAACAGGAACAGGATACACCATGGACACAGTCAACAGAACACATAAATACTCAGAAAAAGGAAAGTGGACAACGAACACAGAGACTGGAGCACCCCAACTCAATCCAATTGATGGACCATTACCTGAGGACAACGAGCCGAGCGGGTATGCACAAACGGATTGTGTATTGGAAGCAATGGCCTTCCTTGAAGAATCTCACCCGGGGATCTTTGAAAATTCGTGTCTAGAAACGATGGAAATTGTTCAGCAAACAAGAGTGGATAAACTGACCCAAGGCCGTCAGACCTATGACTGGACGCTGAATAGAAATCAGCCGGCTGCTACCGCATTGGCCAACACTATAGAAGTATTCAGATCGAATGGCCTAACAGCCAATGAATCAGGAAGGTTGATTGATTTCCTCAAGGATGTGATGGATTCAATGGATAAGGAAGAAATGGAGATTACAACACATTTCCAGAGGAAGAGGAGAGTGAGGGACAACATGACCAAGAAAATGGTCACACAGAGAACAATAGGAAAGAAGAAACAAAGACTGAACAAAAGGAGCTACCTAATAAGAGCACTGACGTTGAACACAATGACAAAGGATGCTGAAAGAGGCAAGCTGAAAAGGAGGGCAATCGCAACACCCGGGATGCAAATCAGAGGATTCGTGTATTTTGTAGAAGCACTAGCGAGGAGCATCTGTGAAAAACTTGAGCAATCTGGCCTCCCTGTCGGAGGGAATGAGAAGAAAGCTAAATTGGCAAATGTTGTGAGGAAGATGATGACTAATTCACAAGATACAGAGCTCTCTTTCACAATTACTGGAGACAACACCAAATGGAATGAAAATCAAAACCCCCGGATGTTTCTAGCGATGATAACATACATCACAAGAAATCAGCCTGAATGGTTTAGAAATGTCTTAAGCATTGCTCCTATAATGTTCTCAAACAAGATGGCGAGATTAGGAAAAGGGTACATGTTCGAAAGTAAGAGCATGAAGTTACGGACACAAGTACCAGCGGAAATGCTCGCAAATATTGACCTGAAATATTTCAACAAATCAACAAGAGAGAAAATCGAGAAAATAAGACCTCTACTGATAGATGGCACAGCCTCATTGAGTCCTGGAATGATGATGGGCATGTTCAACATGTTGAGTACAGTCTTAGGAGTTTCAATTCTGAATCTTGGTCAGAAGAAATACACAAAAACCACATATTGGTGGGACGGACTCCAATCCTCAGATGACTTCGCCCTCATAGTGAATGCACCGAATCATGAGGGAATACAAGCAGGAGTAGATAGGTTCTATAGAACCTGCAAATTAGTTGGGATAAACATGAGCAAGAAGAAATCCTACATAAATAGGACAGGAACATTCGAATTCACAAGCTTTTTCTACCGTTATGGATTCGTAGCTAACTTCAGTATGGAGTTGCCCAGTTTTGGAGTGTCCGGGATTAATGAGTCAGCTGACATGAGCGTTGGTGTTACAGTAATAAAGAACAACATGATAAACAACGATCTTGGACCAGCAACAGCCCAAATGGCCCTTCAGCTATTTATCAAAGACTACAGATACACATACCGATGTCACAGGGGTGACACGCAAATTCAAACGAGAAGAGCATTTGAACTGAAGAAGCTGTGGGAGCAGACCCGCTCGAAGGCAGGACTGTTGGTTTCAGATGGAGGGCCAAACCTGTACAATATTCGGAACCTCCACATTCCAGAGGTCTGCTTGAAATGGGAATTGATGGATGAAGACTACCAGGGCAGGTTGTGTAATCCTATGAACCCGTTTGTCAGTCATAAGGAAATTGATTCAGTAAACAATGCTGTGGTGATGCCAGCTCATGGCCCAGCCAAAAGCATGGAGTATGATGCCGTTGCAACCACACATTCATGGATTCCTAAGAGGAATCGCTCCATTCTCAACACCAGCCAACGGGGGATTCTTGAGGACGAACAGATGTACCAAAAGTGCTGCAACCTATTCGAAAAGTTCTTCCCCAGCAGTTCGTACAGGAGGCCAGTTGGAATTTCCAGCATGGTGGAGGCCATGGTGTCTAGGGCCCGAATTGATGCACGAATTGACTTCGAATCTGGAAGGATTAGGAAAGAAGAGTTTGCTGAGATCATGAAGATCTGTTCCACCATTGAAGAGCTCAGACGGCAAAAATAG

>P5-PB2

ATGGAAAGAATAAAAGAACTAAGAGATTTGATGTCACAGTCTCGCACTCGCGAGATACTGACAAAAACAACAGTGGACCATATGGCCATAATCAAGAAATATACATCAGGAAGACAGGAGAAGAATCCTGCCCTTAGGATGAAGTGGATGATGGCGATGAAATACCCAATCACAGCAGACAAAAGGATAATGGAGATGATCCCGGAAAGAAATGAGCAAGGTCAGACCCTTTGGAGCAAGACAAATGATGCCGGATCAGACAGGGTGATGGTGTCACCTCTGGCTGTGACGTGGTGGAATAGAAATGGACCAACAACAAGTACAATCCATTATCCAAAGGTCTACAAAACCTATTTTGAAAAGGCCGAAAGGCTAAAACATGGAACCTTTGGCCCCGTTCACTTCCGAAACCAAGTTAAAATACGCCGCAGGGTTGACATAAACCCAGGCCATGCAGATCTTAGTGCTAAAGAAGCACAAGATGTCATCATGGAGGTCGTATTCCCAAACGAAGTTGGAGCCAGAATACTGACATCAGAGTCACAGTTAACGATAACCAAGGAAAAGAAGGAGGAGCTTCAGGACTGCAAAATTGCTCCTTTAATGGTGGCATACATGTTGGAGAGAGAACTGGTTCGCAAAACAAGGTTTCTACCAGTGGCTGGAGGGACAAGCAGTGTGTATATCGAAGTATTGCATTTGACCCAAGGGACTTGCTGGGAGCAAATGTACACACCAGGAGGGGGAGTGAGAAATGATGATGTTGATCAGAGTTTAATTATTGCTGCTAGAAATATTGTTAGAAGGGCAACAGTATCAGCAGACCCGTTGGCTTCGCTTTTAGAGATGTGCCATAGTACACAGATTGGAGGGATTAGGATGGTTGACATCCTTAGACAAAACCCAACAGAGGAACAGGCTGTGGATATATGCAAAGCAGCAATGGGTCTAAGGATTAGTTCATCCTTCAGCTTTGGAGGTTTCACTTTCAAAAGGACAAGTGGGTCATCTATCAAAAGGGAAGAAGAAGTGCTCACAGGCAACCTCCAAACATTGAAAATAAGAGTACATGAAGGATATGAGGAATTCACAATGGTTGGGAAAAGAGCAACAGCCATTCTAAGGAAAGCAACCAGAAGACTGATTCAACTGATAGTGAGTGGGAAGGACGAGCAATCAATCGCCGAGGCAATCATAGTGGCAATGGTGTTCTCACAAGAGGATTGTATGATAAAGGCAGTGAGAGGTGATTTGAACTTTGTCAACAGAGCGAACCAGCGGCTAAATCCCATGCATCAACTCCTGAGGCATTTCCAAAAGGATGCAAAGGTCCTGTTTCAAAACTGGGGAATTGAGCCCATTGACAATGTAATGGGGATGATCGGAGTATTACCTGACATGACCCCCAGCACAGAGATGTCTTTGAGAGGAGTGAGAGTTAGTAAAATGGGAGTAGATGAATATTCCAGTACAGAGAGAGTGGTCGTGAGTATTGATCGTTTCTTGAGGGTTCGAGACCAGAGAGGAAACATACTCCTGTCTCCTGAGGAGGTTAGTGAAACACAGGGAACAGAAAGGCTGACTATAACATATTCATCGTCCTTGATGTGGGAAATCAATGGTCCGGAATCAGTGTTAGTTAACACATATCAATGGATCATTAGAAATTGGGAAACTGTAAAGCTTCAATGGTCCCAGGACCCTACAATTCTGTACAATAAGATGGAATTTGAACCCTTTCAATCCCTAGTGCCCAAAGCTGCCAGAGGCCAATATAGTGGATTCGTAAGGGTCCTATTCCAGCAGATGCGTGACGTACTGGGGACGTTCGACACCGTCCAAATAGTAAAGCTACTACCATTTGCAGCAGCCCCGCCGTTCCAGAGTAGGATGCAGTTCTCTTCTCTAACTGTGAACATAAGAGGTTCAGGAATGAGAGTGGTTGTGAGAGGCAATTCTCCAGTGTTCAACTACAATAAGGCAACAAAGAGGCTTACAGTGCTTGGGAAGGATGCAGGTGCGTTGATGGAAGACCCAGACGAGGGAACAGTAGGAGTAGAATCTGCGGTATTGAGAGGATTTCTGATTCTAGGCAAAGAAGACAAAAGATATGGGCCAGCATTGAGCATCAACGAGTTGAGCAACCTTGCGAAAGGGGAAAAGGCTAATGTGTTGATAGGGCAAGGAGACGTGGTGTTGGTAATGAAACGGAAACGGGACTCTAGCATACTTACTGACAGTCAGACAGCGACCAAAAGAATTCGGATGGCCATCAATTAA

>WT-PB1

ATGGATGTCAATCCGACTTTACTTTTCAAAGTGCCAGTACAAAATGCTATAAGTACCACTTTCCCTTATACTGGAGACCCTCCATACAGCCATGGAACAGGAACAGGATACACCATGGACACAGTCAACAGAACACATAAATACTCAGAAAAAGGAAAGTGGACAACGAACACAGAGACTGGAGCACCCCAACTCAATCCAATTGATGGACCATTACCTGAGGACAACGAGCCGAGCGGGTATGCACAAACGGATTGTGTATTGGAAGCAATGGCCTTCCTTGAAGAATCTCACCCGGGGATCTTTGAAAATTCGTGTCTAGAAACGATGGAAATTGTTCAGCACACAAGAGTGGATAAACTGACCCAAGGCCGTCAGACCTATGACTGGACGCTGAATAGAAATCAGCCGGCTGCTACCGCATTGGCCAACACTATAGAAGTATTCAGATCGAATGGCCTAACAGCCAATGAATCAGGAAGGTTGATTGATTTCCTCAAGGATGTGATGGATTCAATGGATAAGGAAGAAATGGAGATTACAACACATTTCCAGAGGAAGAGGAGAGTGAGGGACAACATGACCAAGAAAATGGTCACACAGAGAACAATAGGAAAGAAGAAACAAAGACTGAACAAAAGGAGCTACCTAATAAGAGCACTGACGTTGAACACAATGACAAAGGATGCTGAAAGAGGCAAGCTGAAAAGGAGGGCAATCGCAACACCCGGGATGCAAATCAGAGGATTCGTGTATTTTGTAGAAGCACTAGCGAGGAGCATCTGTGAAAAACTTGAGCAATCTGGCCTCCCTGTCGGAGGGAATGAGAAGAAAGCTAAATTGGCAAATGTTGTGAGGAAGATGATGACTAATTCACAAGATACAGAGCTCTCTTTCACAATTACTGGAGACAACACCAAATGGAATGAAAATCAAAACCCCCGGATGTTTCTAGCGATGATAACATACATCACAAGAAATCAGCCTGAATGGTTTAGAAATGTCTTAAGCATTGCTCCTATAATGTTCTCAAACAAGATGGCGAGATTAGGAAAAGGGTACATGTTCGAAAGTAAGAGCATGAAGTTACGGACACAAGTACCAGCGGAAATGCTCGCAAATATTGACCTGAAATATTTCAACAAATCAACAAGAGAGAAAATCGAGAAAATAAGACCTCTACTGATAGATGGCACAGCCTCATTGAGTCCTGGAATGATGATGGGCATGTTCAACATGTTGAGTACAGTCTTAGGAGTTTCAATTCTGAATCTTGGTCAGAAGAAATACACAAAAACCACATATTGGTGGGACGGACTCCAATCCTCAGATGACTTCGCCCTCATAGTGAATGCACCGAATCATGAGGGAATACAAGCAGGAGTAGATAGGTTCTATAGAACCTGCAAATTAGTTGGGATAAACATGAGCAAGAAGAAATCCTACATAAATAGGACAGGAACATTCGAATTCACAAGCTTTTTCTACCGTTATGGATTCGTAGCTAACTTCAGTATGGAGTTGCCCAGTTTTGGAGTGTCCGGGATTAATGAGTCAGCTGACATGAGCGTTGGTGTTACAGTAATAAAGAACAACATGATAAACAACGATCTTGGACCAGCAACAGCCCAAATGGCCCTTCAGCTATTTATCAAAGACTACAGATACACATACCGATGTCACAGGGGTGACACGCAAATTCAAACGAGAAGAGCATTTGAACTGAAGAAGCTGTGGGAGCAGACCCGCTCGAAGGCAGGACTGTTGGTTTCAGATGGAGGGCCAAACCTGTACAATATTCGGAACCTCCACATTCCAGAGGTCTGCTTGAAATGGGAATTGATGGATGAAGACTACCAGGGCAGGTTGTGTAATCCTATGAACCCGTTTGTCAGTCATAAGGAAATTGATTCAGTAAACAATGCTGTGGTGATGCCAGCTCATGGCCCAGCCAAAAGCATGGAGTATGATGCCGTTGCAACCACACATTCATGGATTCCTAAGAGGAATCGCTCCATTCTCAACACCAGCCAACGGGGGATTCTTGAGGACGAACAGATGTACCAAAAGTGCTGCAACCTATTCGAAAAGTTCTTCCCCAGCAGTTCGTACAGGAGGCCAGTTGGAATTTCCAGCATGGTGGAGGCCATGGTGTCTAGGGCCCGAATTGATGCACGAATTGACTTCGAATCTGGAAGGATTAGGAAAGAAGAGTTTGCTGAGATCATGAAGATCTGTTCCACCATTGAAGAGCTCAGACGGCAAAAATAG

>WT-PB2

ATGGAAAGAATAAAAGAACTAAGAGATTTGATGTCACAGTCTCGCACTCGCGAGATACTGACAAAAACAACAGTGGACCATATGGCCATAATCAAGAAATATACATCAGGAAGACAGGAGAAGAATCCTGCCCTTAGGATGAAGTGGATGATGGCGATGAAATACCCAATCACAGCAGACAAAAGGATAATGGAGATGATCCCGGAAAGAAATGAGCAAGGTCAGACCCTTTGGAGCAAGACAAATGATGCCGGATCAGACAGGGTGATGGTGTCACCTCTGGCTGTGACGTGGTGGAATAGAAATGGACCAACAACAAGTACAATCCATTATCCAAAGGTCTACAAAACCTATTTTGAAAAGGCCGAAAGGCTAAAACATGGAACCTTTGGCCCCGTTCACTTCCGAAACCAAGTTAAAATACGCCGCAGGGTTGACATAAACCCAGGCCATGCAGATCTTAGTGCTAAAGAAGCACAAGATGTCATCATGGAGGTCGTATTCCCAAACGAAGTTGGAGCCAGAATACTGACATCAGAGTCACAGTTAACGATAACCAAGGAAAAGAAGGAGGAGCTTCAGGACTGCAAAATTGCTCCTTTAATGGTGGCATACATGTTGGAGAGAGAACTGGTTCGCAAAACAAGGTTTCTACCAGTGGCTGGAGGGACAAGCAGTGTGTATATCGAAGTATTGCATTTGACCCAAGGGACTTGCTGGGAGCAAATGTACACACCAGGAGGGGGAGTGAGAAATGATGATGTTGATCAGAGTTTAATTATTGCTGCTAGAAATATTGTTAGAAGGGCAACAGTATCAGCAGACCCGTTGGCTTCGCTTTTAGAGATGTGCCATAGTACACAGATTGGAGGGATTAGGATGGTTGACATCCTTAGACAAAACCCAACAGAGGAACAGGCTGTGGATATATGCAAAGCAGCAATGGGTCTAAGGATTAGTTCATCCTTCAGCTTTGGAGGTTTCACTTTCAAAAGGACAAGTGGGTCATCTATCAAAAGGGAAGAAGAAGTGCTCACAGGCAACCTCCAAACATTGAAAATAAGAGTACATGAAGGATATGAGGAATTCACAATGGTTGGGAAAAGAGCAACAGCCATTCTAAGGAAAGCAACCAGAAGACTGATTCAACTGATAGTGAGTGGGAAGGACGAGCAATCAATCGCCGAGGCAATCATAGTGGCAATGGTGTTCTCACAAGAGGATTGTATGATAAAGGCAGTGAGAGGTGATTTGAACTTTGTCAACAGAGCGAACCAGCGGCTAAATCCCATGCATCAACTCCTGAGGCATTTCCAAAAGGATGCAAAGGTCCTGTTTCAAAACTGGGGAATTGAGCCCATTGACAATGTAATGGGGATGATCGGAGTATTACCTGACATGACCCCCAGCACAGAGATGTCTTTGAGAGGAGTGAGAGTTAGTAAAATGGGAGTAGATGAATATTCCAGTACAGAGAGAGTGGTCGTGAGTATTGATCGTTTCTTGAGGGTTCGAGACCAGAGAGGAAACATACTCCTGTCTCCTGAGGAGGTTAGTGAAACACAGGGAACAGAAAGGCTGACTATAACATATTCATCGTCCTTGATGTGGGAAATCAATGGTCCGGAATCAGTGTTAGTTAACACATATCAATGGATCATTAGAAATTGGGAAACTGTAAAGCTTCAATGGTCCCAGGACCCTACAATTCTGTACAATAAGATGGAATTTGAACCCTTTCAATCCCTAGTGCCCAAAGCTGCCAGAGGCCAATATAGTGGATTCGTAAGGGTCCTATTCCAGCAGATGCGTGACGTACTGGGGACGTTCGACACCGTCCAAATAGTAAAGCTACTACCATTTGCAGCAGCCCCGCCGGAACAGAGTAGGATGCAGTTCTCTTCTCTAACTGTGAACATAAGAGGTTCAGGAATGAGAGTGGTTGTGAGAGGCAATTCTCCAGTGTTCAACTACAATAAGGCAACAAAGAGGCTTACAGTGCTTGGGAAGGATGCAGGTGCGTTGATGGAAGACCCAGACGAGGGAACAGTAGGAGTAGAATCTGCGGTATTGAGAGGATTTCTGATTCTAGGCAAAGAAGACAAAAGATATGGGCCAGCATTGAGCATCAACGAGTTGAGCAACCTTGCGAAAGGGGAAAAGGCTAATGTGTTGATAGGGCAAGGAGACGTGGTGTTGGTAATGAAACGGAAACGGGACTCTAGCATACTTACTGACAGTCAGACAGCGACCAAAAGAATTCGGATGGCCATCAATTAA
