# Supplementary material for: Risk of Environmental Exposure to H7N9 Influenza Virus via Airborne and Surface Routes in a Live Poultry Market in Hebei, China
Source: Front Cell Infect Microbiol. 2021 Jun 7;11:688007. doi: 10.3389/fcimb.2021.688007 (PMC8216215; doi:10.3389/fcimb.2021.688007)
Supplement: Supplementary file 2 [file Table_1.docx]

| Segment | Position | WT | P1 | P2 | P3 | P4 | P5 |
| --- | --- | --- | --- | --- | --- | --- | --- |
| PB1 | 343-345 | CAC | CAA | CAA | CAA | CAA | CAA |
| PB2 | 1879-1881 | GAA | TTC | TTC | TTC | TTC | TTC |

SUPPLEMENT TABLE 1. Nucleotide substitution in the mouse-adapted H7N9 influenza virus

WT virus and all passaged H7N9 viruses have been sequenced. All Substituted nucleotides were listed on the table.
